# Integrating Energy and Environmental Management in Wood Furniture Industry

**DOI:** 10.1155/2014/596958

**Published:** 2014-01-21

**Authors:** Dušan Gordić, Milun Babić, Dubravka Jelić, Davor Konćalović, Vladimir Vukašinović

**Affiliations:** Faculty of Engineering, University of Kragujevac, Sestre Janjić 6, 34000 Kragujevac, Serbia

## Abstract

As energy costs continue to rise, industrial plants (even those of energy nonintensive industries such as furniture industry) need effective way to reduce the amount of energy they consume. Besides, there are a number of economic and environmental reasons why a company should consider environmental management initiatives. This paper provides a detailed guideline for implementing joint energy and environmental management system in wood furniture industrial company. It covers in detail all essential aspects of the system: initial system assessment, organization, policy development, energy and environmental auditing, action plan development, system promotion, checking system performance, and management review.

## 1. Introduction

The wood furniture industry includes manufacturing of furniture parts and their assembly with appropriate finishing operations. Basic materials in the industry are wood and wood-based materials (plywood, hardboard, MDF, HDF, OSB, etc.). Other materials, such as metal, foam, cloth, and plastic, are also used.

The furniture manufacturers predominantly belong to the group of small and medium companies. The level of specific energy consumption (energy consumed per unit of finished product) depends on the manufacturing processes that are implemented in a company, the type of material being processed, applied woodworking technology, scale and type of production, the type of furniture that is produced, and so forth. Furniture industry is relatively small energy consumer according to the classifications of DOE (US Department of Energy) and IEA (International Energy Agency). According to the systemization of data for different industries, the mean specific consumption of primary energy in the wood processing industry (which includes the production of wood furniture) in the EU is 0.0332 toe/t of finished product [1]. Although the amount is relatively small, the potential for energy savings exists.

Energy costs in an energy nonintensive industrial company (such as a furniture industry company) are often considered as fixed overheads, although they belong to the cost categories that can be easily managed. However, due to the constant growth of energy prices, industrial companies in the industry become increasingly aware that the energy is an expensive commodity and therefore it should be used effectively in order to increase company's profit. Therefore, companies become more motivated to introduce the practice of energy management as a part of their regular activities [2, 3].

Until recently, energy management practice primarily considered replacing inefficient equipment and then using any number of methods to assess obtained savings. Experience shows that positive effects of energy-efficient improvements were decreased over time. This is due to the fact that energy use in an industrial company is largely dependent on operational practices. Production volume, schedule of operations, or the type of products that is being produced in an industrial plant can be significantly changed during the life of the company. Energy consumers in production process can be relatively energy efficient in initial production scenario, but significantly less effective under the changed production conditions. The presence of energy-efficient equipment, although very important, does not ensure that the industrial system will be energy efficient. It is often the case that the energy-efficient equipment is being used in nonenergy-efficient way in some companies [4].

Energy management is an ongoing process which includes the monitoring energy performance and constantly finding ways to improve and maintain the performance. There have been significant efforts during the last decade to define appropriate standards and best practices and implement the consistent energy management system to increase and maintain the energy savings. Knowledge and experience gained in the implementation of the thousands of energy efficiency projects caused the shift from traditional tactical level (once “build and forget” projects) to the strategic level of energy management, proposed and supported by a number of relevant international organizations, including the Energy Star (USA), National Resources Canada (Canada), Action Energy (UK), and EPA Victoria (Australia). Several national energy management standards exist: Denmark—DS/INF 136, Ireland—IS 393:2005, Sweden—SS 627 750, USA—ANSI MSE 2000, and so forth [5]. Moreover, CEN - European Committee for Standardization, published European Standard EN 16001:2009 in July 2009 and the new International Standards Organization Management System Standard for Energy—ISO 50001—was published on 15th July 2011.

Energy management system is focused on the efficient use of energy, water, and other raw materials. Standard activities in a systematic approach to energy management include energy procurement, billing and measurement, performance measurement, development of energy policy, energy auditing, establishing a correlation between energy used and the volume of final production, raise awareness, implementation of energy efficiency projects, training and education, management of investment projects, and so forth [3, 6].

The main environmental concerns of the wood-based furniture industry include the following:

- air pollution from sawdust, other particulates, and VOCs;
- water pollution from used solvents and other spent finishing materials as well as from maintenance and clean-up operations and
- solid wastes comprising of wood chips, sawdust, adhesive and resin particles, and general trash (Table 1).

The magnitude of these concerns depends largely on the size of the manufacturing operations and the nature and degree of technological sophistication of the equipment and facilities being employed. The factory workers are the first to be affected by the emissions produced by various operations, but surrounding areas can also be adversely impacted in the absence of minimum preventive controls.

It should be also emphasized that furniture manufacturing companies often lack accounting evidence on generated wood waste [7].

To improve the environmental performance of a company, it is often necessary to implement expensive measures without obvious and direct financial benefits for the company. Therefore, environmental issues were not on the priority list of industrial companies. However, furniture manufacturers are more faced with strict environmental regulations and restrictions which cause their need for environmental management. Besides, the proper attitude towards the environment of an industrial company is a desirable corporate behaviour which significantly affects the good image of the company at consumers and competition.

Continual environmental pollution, fear of complete exhaustion of natural resources, increased public interest for preserving the environment, lack of organized and systematic monitoring of pollution consequences, and specific working conditions in the affected areas have led to the obvious need for the introduction of environmental management systems that are defined by standards, such as international ISO 14001:2004 and the EU - EMAS (Eco-Management and Audit Scheme) [8, 9].

An environmental management system should take into account the legislation, existing standards, and being consistent with the needs of the environment. It should control and reduce pollution and impact of the pollution on the environment originated from using raw materials and energy in production.

Benefits from applying the system in the industry are

- reducing the environmental impact,
- reducing the risk of potential environmental disasters, and
- reducing the costs required to maintain the system in accordance with the environment.

Environmental protection costs are the consequence of

- investment and operating costs for the equipment of pollution control system,
- tariff for the disposal of waste (hazardous materials),
- training required for the environmental protection,
- monitoring, storing and analyzing data, reporting, and so forth.

Under the system, environmental policy is defined and implemented, objectives and expectations are set, and the system for monitoring of environmental impacts arising as a result of production in an industrial company is established. The procedures to ensure continuous improvement of the impact of company performance on the environment and the procedures to reduce current and avoid future environmental accidents are also implemented.

The energy management system should equally take into account the cost of energy and the efficiency of transformation, generation, and distribution of energy. On the other hand, the environmental management system is primarily focused on: compliance with legislation, minimization of the impact of production on the environment and reduction of emissions to atmosphere, and generation of solid waste and wastewater. Both management systems deal with the efficiency of raw material usage and waste reduction, since any (unnecessary) waste of raw materials means that more than necessary energy is being used and that environmental pollution is greater than necessary.

According to the analysis of relevant research, individual elements of the energy management system can be compared with the elements of the environmental management system [1, 10]. In an industrial company, universal practice of joint management of energy and environment can be therefore introduced. This enables continuous improvement of the energy and environmental performance of a plant and entire company with the primary aims of reducing: operational costs (achieved through energy savings and waste disposal costs), the amount of generated waste, and the impact of the company on the environment.

## 2. Energy and Environmental Management System

The energy and environmental management system (EEMS) presents a system for improving energy and environmental performance of a company. Practically, it is a system that should enable the achievement of established goals by measuring, monitoring, and evaluating energy and environmental performance of production processes in industrial companies [11].

More specifically defined, EEMS integrates the following elements:

- people with the necessary skills and assigned responsibilities,
- published policy with clear and transparent objectives,
- defined procedures for its implementation,
- established measurement system for performance monitoring,
- action plan for continuous improvement,
- reporting system to check progress and monitor results,

with primary goal of achieving continuous energy and environmental performance improvement [1].

The benefits of integrating systems of energy management and environmental management are

- avoidance of parallel management systems for energy and environmental protection,
- less administration in the company,
- easier maintenance of the system,
- greater financial savings and less environmental pollution [10].

The main disadvantage of integrating energy and environmental management systems is the fact that it becomes large, overly complex and it is not suitable for the organization and the current responsibilities. According to [12] furniture manufacturers are industry where a low degree of energy management or no energy management exists, so practically there are no significant obstacles in introducing EEMS to their practice.

Each phase of EEMS introduction can be viewed as a process which requires time, material, and human resources. It is important to bear in mind that this is a system that achieves continuous improvement. The system takes care of both technical and human aspects of activity in the company, requires the constant support of top management, high-quality employees in energy and environmental management departments, and permanent source of funding.

**Figure 1:**
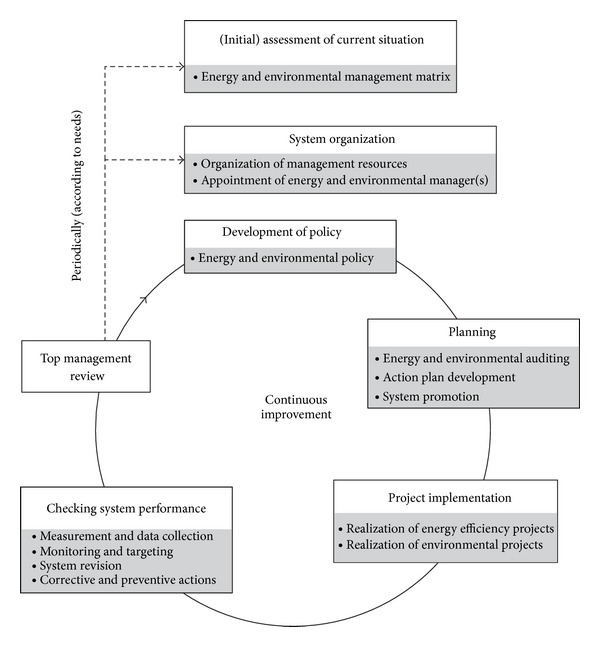
Structure of energy and environmental management system (EEMS).

EEMS in an industrial company depends on type of industry, products, production volume, level of automation, number of workers, condition of production equipment, and so forth. But no matter what the type of a company is, the basic structural elements of the system are shown in Figure 1. The elements of EEMS in a wood furniture production company are presented in the following text.

## 3. Elements of Energy and Environmental Management System

### 3.1. Energy and Environmental Management Matrix

Before starting the implementation of EEMS in a company, it is recommended to analyze the current state of energy management and its impact on the environment. Even later, during EEMS development and its implementation, it is useful to periodically analyze the situation, to see where the company is and what needs to be corrected and to be done to achieve the desired [13]. Energy and environmental management matrix presents an effective way to gain insight into a current approach to energy and environmental matters in the company (Table 2). Each column of the matrix deals with one of the six most important aspects of energy and environmental management:

- company policy towards energy management and environmental protection,
- organising,
- staff motivation,
- information system for tracking, monitoring, and reporting,
- awareness, training, and promotion of employees, and
- investments.

The matrix identifies those aspects where some further attention is required to ensure that EEMS is developed in an effective way. The most common situation in a company is that levels of implementation of energy management and environmental management are different. The aim of a company should be to evenly move to higher levels, with the balance in each column in both aspects. Regular matrix reviews enable to notice and publish the progress in EEMS.

### 3.2. Organising EEMS

The decision of management to control energy costs and costs of harmonization with environmental regulatory requirements presents an important first step in starting any kind of energy and environmental management. It cannot achieve a lot without management decision and therefore this decision must be made clear to employees. When the top management is dedicated to energy and environmental management, it is important to define and document the respective roles, responsibilities, authorities, and interrelated functions and to reallocate personnel and financial resources for EEMS implementation. It is necessary to issue and distribute a support document in written, so that all employees are aware of the nature of the proposed measures and to know who is responsible for their implementation. The document should also clearly state that everyone should be aware of wasting energy and generating too much waste and that the obligation of employees is to contribute to reducing waste in all its forms [14].

Top management should share responsibilities and encourage the free participation of employees at all levels. If a sense of “ownership” is not encouraged, the chances of success are limited. Regardless of the expensive measurement equipment, applied technological solutions, and a modern monitoring system, an EEMS will not be successful if employees do not feel the need to participate in solving problems related to excessive energy consumption and environmental protection. It is necessary to convince employees to change their attitude towards the problems of energy saving and environmental protection.

An important part of top management commitment is establishing organization responsible for EEMS implementation. It usually consists of two levels:

- energy and environmental managers and
- energy and environmental committee.

Approved resources, level of support, and authorities assigned to the managers and the committee can be seen as the evidence of top management commitment.

The existence of leaders that will tirelessly seek opportunities to improve energy and environmental performance of a company is essential for the successful implementation of EEMS. These individuals—energy and environmental managers—must sincerely believe in necessities and opportunities for improving performance. These managers are driving force of the system and as program leaders they must have sufficient personal and professional authority in order to be respected, listened, and followed by employees. They are partly strategist, partly project managers, and partly coordinators of changes. They should be able to focus on the technical aspects of their job and also to focus on problems of communication, motivation, and involvement of employees. Most often energy and environmental managers are selected among employees. The advantage of this approach lies in the fact that these individuals are familiar with the technological aspects, organizational structure, and personnel.

The role of energy and environmental managers varies from company to company, but these people are mainly responsible for

- developing energy and environmental policy,
- regularly collecting and analyzing data related to energy consumption and to the amount of generated emissions of gaseous, liquid, and solid materials from the production process,
- monitoring the supply of energy and raw materials that can cause a negative impact on the environment, health, and safety of workers,
- researching and identification of best practices in energy and environmental management,
- identifying opportunities for energy saving and reducing waste generation,
- developing energy efficiency and environmental protection projects (including necessary technical and economic assessment),
- implementing energy efficiency and environmental protection projects,
- maintaining communication (with senior management and employees of a lower hierarchical level),
- reporting to senior management and publishing the success of employees,
- EEms promoting and public relations [1, 14, 15].

Depending on the size and activities of a company, one or more managers for energy and usually one or more managers for the environment are chosen. Since the furniture industry is energy non-intensive industry and companies in this industry belong to the group of small and medium ones, it is recommended to choose a person—energy and environmental manager, who will be obliged to take care of environmental protection and energy efficiency in the company. Energy and environmental manager, if necessary, establishes team (group) of coordinators for energy and environmental management. This group should be appointed for certain period of time (usually one year). Rotating members bring new people with fresh ideas creating a mechanism for a smooth replacement of inactive members and providing constructive involvement of staff. Team members should be chosen to have the skills that manager lacks. While the energy and environmental manager has full-time engagement, team members will be engaged only on part-time basis. For larger companies with more plants and complex equipment, team members can be engaged on a full-time basis.

In many companies (especially large, from energy-intensive industries, and with substantial influence of the manufacturing process on the environment) the appropriate committee is formed. This committee is a team of enthusiasts formed to help energy and environment manager(s) in the process of introduction of EEMS. This group should be retained even after the implementation phase to coordinate and make regular EEMS assessment.

Employees are the last level of the organizational structures. As was already mentioned, they are the most inexhaustible source of energy and environmental management program. Good energy and environmental manager will allocate up to 20% of his time to work with employees because workers in manufacturing plants know much more about equipment than anyone in the plant, since they are directly addressed to it. They know how to manage energy more effectively and efficiently use resources, but if there is no mechanism that would encourage them to act, their ideas will remain unrealized. For the successful involvement in energy and environmental management program, employees should be motivated because they support the program provided that they are encouraged, feel needed and important, will be allowed to stand out, are credited for their achievements, are helped to enjoy their work, rather than to be bored, are offered a more interesting and responsible jobs, are continuously informed about how their ideas and efforts were beneficial, and are rewarded (financially and otherwise) for contribution to energy and environmental efficiency.

Besides communication, an important factor for maintaining the motivation in a company is the education of employees. Adequate knowledge and skills for the proper fulfilment of the tasks of all employees involved in EEMS are ensured with appropriate training. There are two types of training that directly contribute to the efficient use of energy and raw materials:

- awareness raising training which should give to everyone in the organization stimulus, motivation, and confidence that being more energy efficient and environmentally responsible is the correct approach,
- training about resources and technology which provides adequate resources and technical knowledge for the work performance of the staff in energy-efficient and environmentally responsible manner.

Besides these trainings, the education may consist of courses with indirect effects (courses in communication, social and organizational skills, trainings about project management, etc…).

Depending on the current situation in a company, it is possible that the awareness raising training is undertaken at early stage of EEMS implementation. After defining the company needs and action plan for EEMS implementation, the training is followed by trainings about tools and technology [15].

### 3.3. Development of Energy and Environmental Policy

Once senior management decides to introduce EEMS the attention is paid to the development of energy and environmental policy. This policy is a document that expresses company's commitment to increase energy efficiency, reduce negative environmental impact, and increase safety and health of workers.

There are several reasons why a company benefits from adopting an official energy and environmental policy:

- a clear statement will give a sense of purpose, increasing the chances of success,
- senior management can assess the impact of the strategy in relation to agreed set of goals,
- the need for energy efficiency and environmental protection is better understood and accepted within the company if the support of senior management exists,
- activities will be successful if energy and environmental management is allocated with adequate resources,
- it is an opportunity to “put on paper” obligations and responsibilities for consumed energy and generated waste in the company.

Energy and environmental policy sets targets for the required level of performance of a company concerning energy consumption and waste generation. All following actions will be assessed with respect to the targets. The policy defines the responsibilities for energy efficiency and environmental protection in a company. It is an imperative to formally adopt written document of energy and environmental policy at the board of directors or other equivalent level. The policy will not have the necessary management support without formal adoption [16].

Defining the energy and environmental policy is a task to which every company approaches in a unified way emphasizing aspects that are the most important for its own business. Since the energy costs in wood furniture industry are relatively small (a few % of total company's costs) energy and environmental policy should be focused on efficient use of raw material and productivity improvements through waste minimization and reuse of waste material as energy source. Thus the example of energy and environmental policy in a wood furniture company should contain the information shown in Table 3.

### 3.4. Energy and Environmental Auditing

In order to identify opportunities to reduce energy, water, and raw material costs and costs of harmonization of company's environmental performance with regulatory requirements, it is necessary to implement energy and environmental auditing. The auditing involves recording of current state and balancing of energy and material flows. It may be related to a technological process, production line, plant, or whole company. Auditing is used to estimate currently (base) state of energy and environmental efficiency of the company, in respect of which the company's energy and environmental performance will be monitored during the EEMS implementation.

Energy and environmental auditing involves a study on the current state of EEMS in industrial plants which should

- determine the consumption of all energy used in the company and all types of waste generated in the production plants,
- identify the major energy consumers and determine their share in total energy consumption of the company,
- identify locations in the production process with the largest negative impact on the environment, safety, and health of employees,
- identify legal and other requirements imposed on the company in relation to environment, safety, and health of employees,
- identify the most cost-effective measures to improve energy and environmental efficiency of the company,
- assess the potential of energy savings, implementation costs, and payback period for the proposed measures or actions to increase energy efficiency,
- assess the potential of decreasing negative impacts on environment, safety, and health of employees, as well as cost of implementation and possibly the payback period for the proposed measures and activities to increase environmental efficiency,
- review the energy and environmental policy, including evaluation system on effects of implemented measures.

Energy and environmental auditing is performed by the staff—energy and environmental manager with their team, if they have sufficient knowledge, skills, and resources for the analysis of the balancing of energy flows and matter, or external consultants may be engaged. The engagement of external consultants is recommended because these people are independent; they have a broader point of view and they know the most modern technologies.

According to a defined scope and purpose, there are two types of audits: *preliminary* (considers initial data collection to evaluate the current state based on a review of existing documentation and a brief overview of a company, during which the audit team inspects the general condition of a equipment, maintenance standards, the level of operational control, and reporting procedures) and *detailed* (includes a comprehensive recording and analysis of flows of energy and material in the company. Measuring instruments are commonly used to check the way of energy is used and to determine the quantity of waste material which is accompanied with detailed analysis of various systems and subsystems) [17–19].

In practice, there are many cases where the energy and environmental audit is between those two, more complex than a preliminary, but not as extensive as the detailed. Work on audit requires a flexible approach in which procedures are adapted to the needs of the company.

The energy and environmental auditing includes the collection of various data:

- general information (information about the type of industry, the projected production volume, number of working hours, equipment specifications with accompanying technical data, technological schemes and description of plants, plant dispositional drawings, etc.)
- historical data on energy consumption and energy costs (data from energy bills) and the monthly production volume,
- historical environmental data (data on quantities of disposed solid waste, permit to discharge wastewater into the sewer or controlled water flows, data on emissions into the air, records of complaints and environmental incidents, the details of violations of environmental regulations and related legal processes, etc.)
- data on the possible generation of energy (self-production of hot water, steam, electricity),
- data from measuring devices for measurement of energy parameters and quantities of generated waste,
- information obtained by interviewing employees (technical staff, operators, and maintainers) about problems associated with increased energy consumption and significantly increased influence on the environment and occupational safety and health.

**Figure 2:**
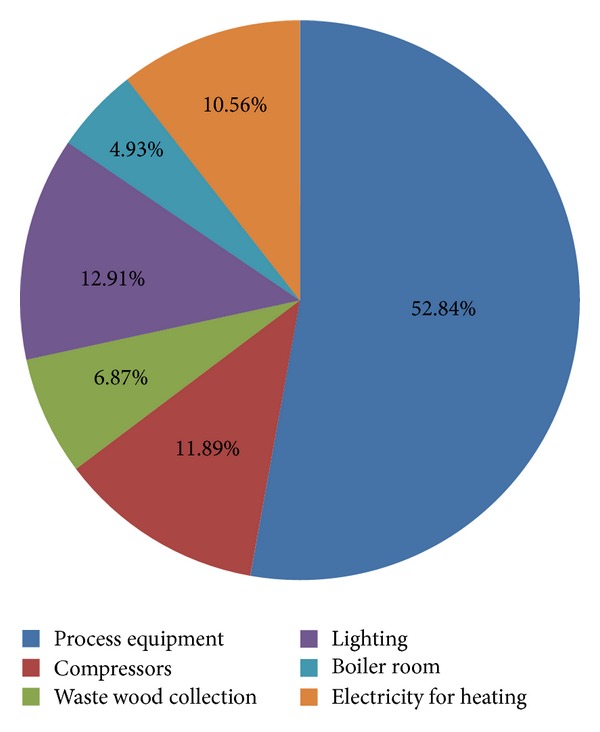
Distribution of electricity consumption in a wood furniture industry company.

Collected data should be systematized, analyzed, and presented in a way (tabular or graphic) which allows evaluation of options for saving energy and reducing the negative environmental impact of production. Some examples showing the data obtained in energy and environmental auditing in a wood furniture industry company are shown in Tables 4 and 5 and Figure 2.

**Figure 3:**
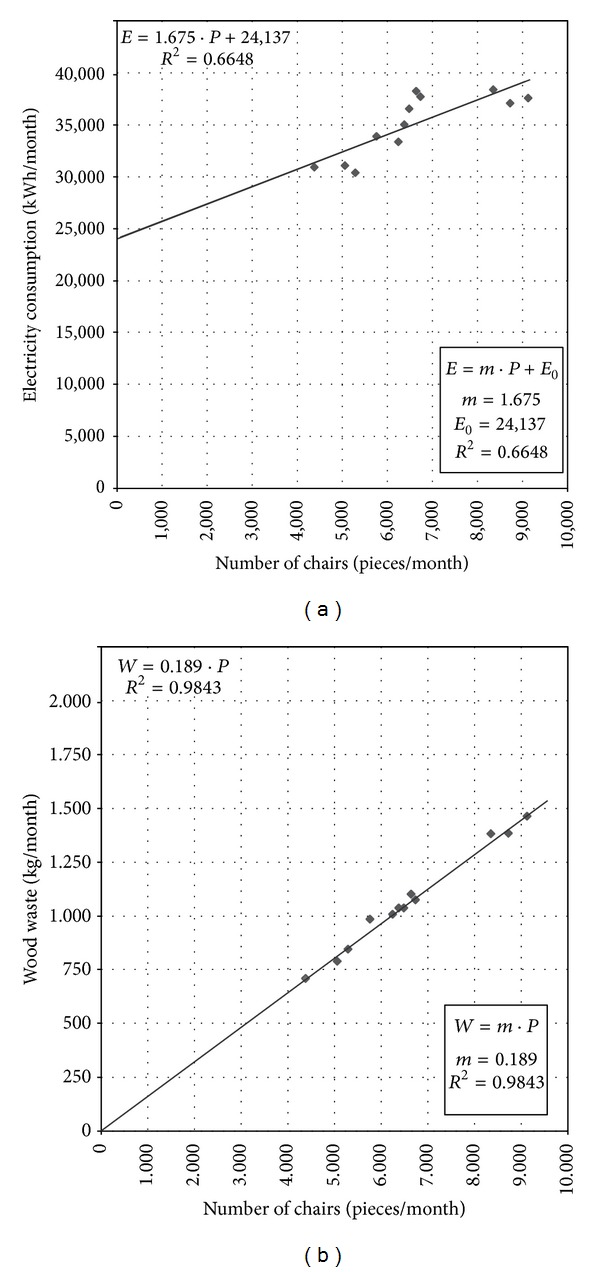
Dependence of (a) energy consumption (b) generated waste on production volume.

For the analysis, diagrams of energy consumption—*E* (the amount of waste materials—*W*) in terms of production volume—*P* should be created for each energy source and each waste material. These functional dependences are close to a straight line to a common industrial plant (Figure 3):

(1) E=m·P+E0 i.e.,  W=m·P+W0,where  as  a  rule  W0=0.

There are three basic elements which should be analyzed in the diagram:

- Ordinate intercept—*E*_0_, which shows how much energy is consumed when the process takes place without any production. It is also the energy consumed in production, but it does not contribute to production (e.g., for electricity: lighting, electricity for office equipment, circulation pumps, compressors, fans, electricity for plant maintenance, energy for equipment idling);
- Slope—*m*, which represents the amount of energy consumed (the amount of waste generated) at a given level of production to process each additional unit of production. The efficiency of the process can be established from the slope (if the slope is less, the situation is better);
- Coefficient of determination—*R* ^2^, describing the degree of points' scatter which takes the values 0 ≤ *R* ^2^ ≤ 1. It is a general indicator of energy management (waste management) in a company. Widely scattered points (*R* ^2^ close to 0) usually indicate that the processes are not adequately controlled and that operational practice is inadequately defined and poorly managed by those responsible.

Energy and environmental auditing is used for defining energy and environmental performance indicators. Energy performance indicators (energy efficiency indicators) are expressed in the form of specific energy consumption, defined as the ratio of energy consumption (in physical units or monetary equivalent of the cost of supply) to the production output (or less common to the quantity of input material). Environmental performance indicators are expressed as the ratio of the quantity of gaseous, liquid, or solid waste to the amount of used resource (raw materials or energy).

**Figure 4:**
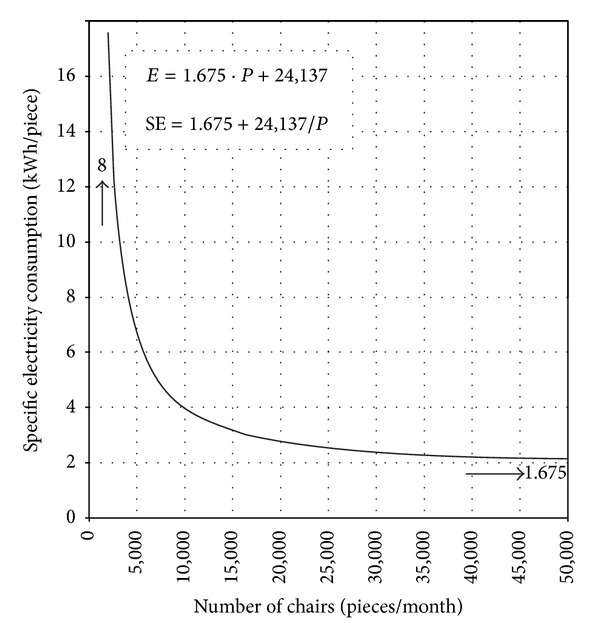
Dependence of specific energy consumption on production volume.

Data on specific energy consumption does not mean much until they are related to production volume or to the level of company's capacity utilization. This is especially true for companies in which the volume of production varies considerably over time. Therefore, apart from analysis of energy consumption related to production volume, the audits should show the dependence of specific energy consumption—SE on production volume (Figure 4):

(2) $$\begin{array}{c} \text{SE}=m+\frac{E_{0}}{P}. \end{array}$$

**Figure 5:**
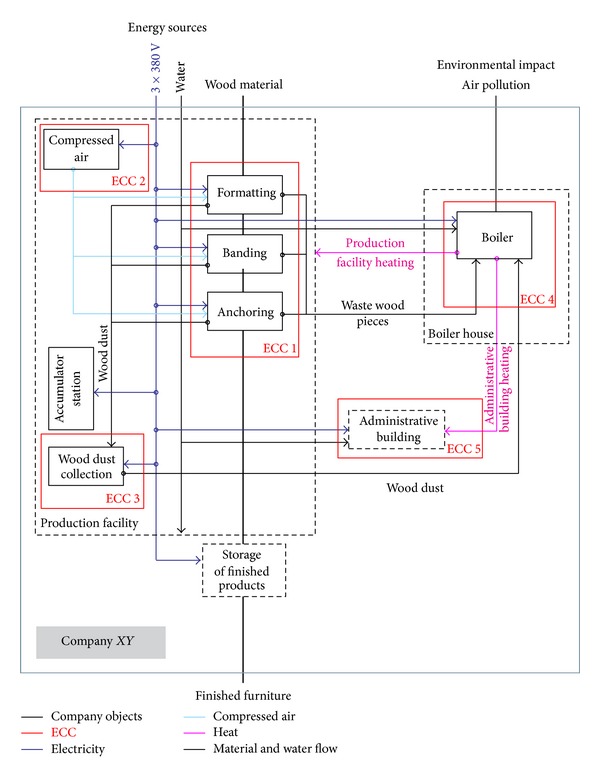
ECCs in a company XY which produces furniture from wood-based board materials.

EEMS involves the decentralization of responsibilities for energy and environmental performance along the production lines and energy flows. It considers defining centres responsible for energy consumption and negative environmental impact. These business units (plants, departments, group of equipment, or single equipment) that use significant amounts of energy or create significant environmental impact are called energy cost centres—ECC [1]. An example of defining and schematic representation of ECC in the one company that produces furniture from wood-based board materials is shown in Figure 5.

After careful review of the industrial company and energy and environmental auditing, the next step is defining the measures and activities (with cost-effectiveness analysis for each intervention) that are later included in the report with recommendations for company's management. Measures and activities to save energy and increase the environmental efficiency of the company are identified by comparative statistics, experience, and best practice. The measures and activities may be classified according to the level of funding necessary for their implementation (no cost and low-cost measures and activities, medium investment measures, and high capital investment measures).

Measures and activities to increase energy efficiency in the wood furniture industry are presented in Table 6, and the measures and activities to increase environmental efficiency are presented in Table 7.

The results of energy and environmental auditing should be systematized in the form of clear and concise reports. No matter how comprehensive and well-performed energy and environmental audit was, its value depends on the quality of the final report. Some of the key elements of the report are

- abstract (summary) with recommendations listed by priority with cost estimations for their implementation and payback period,
- relevant information about the facility and production process,
- information about equipment, measuring data, or assessment of energy consumption and quantity of waste generated for each part of the plant,
- data and graphical analysis of data on energy consumption and the quantity of generated waste,
- details of energy and environmental efficiency improvement,
- comparison of current energy consumption or waste generation with anticipated consumption (generation) (after the implementation of recommended measures and activities),
- recommendations for the inclusion of energy and environmental strategy such as monitoring systems and regular reviewing [14, 15, 20].

The main goal of the final report is to convince company's management to invest in the proposed measures and activities that will increase energy and environmental efficiency.

### 3.5. Action Plan Development

The true value of energy and environmental auditing is given only when the recommendations from the report are included in an action plan of EEMS in a company. As a result of auditing report, different types of initiatives could arise

- changes in operating procedures,
- revision of maintenance system, because it affects the efficient use of energy and raw materials,
- modification or replacement of existing plant and/or equipment,
- subsequent detailed studies on the reduction of energy consumption of a plant or process and
- commitment to continuous training and information dissemination in order to increase awareness of employees about energy saving and environmental protection issues.

Energy and environmental managers (along with their team) should gather all the possibilities for improvement and set up a system for transparent and comprehensive procedure for the selection of measures to be applied based on auditing reports. This ensures the selection of the best solutions and support of employees in planned activities implementation.

The action plan should contain the following items:

- defined measures and activities to increase energy and environmental efficiency and criteria for their selection,
- details of responsibilities and allocation of resources (human, technical, financial) for plan implementation,
- relevant legal obligations and defined objectives of the energy and environmental performance and timeline to achieve the objectives,
- approved reporting procedures for monitoring energy consumption and the amount of waste and associated costs,
- regular review of the action plan,
- defined procedures to include improvements.

The action plan should be regularly updated and improved based on lessons learned from the system implementation. Energy and environmental policy of management should also be redefined to include modifications of action plan targets [15, 20].

### 3.6. EEMS Promotion

Successful EEMS requires commitment and effort of employees at all levels in business organisation. To ensure accountability and resources, it is necessary to

- identify internal and external resources that can help in the implementation and
- integrate EEMS in the work plans and organizational structure in order to secure that resources are available.

Employees have to accept their responsibilities and fulfil the tasks thus contributing to optimal energy and environmental management. Each individual should constantly be aware of the importance of assigned role. The awareness that there are potentials for energy savings and increasing environmental efficiency, a clear job description, unambiguous work instructions, and additional trainings are vital to the success of energy and environmental management.

Moreover for the successful implementation of EEMS it is necessary for individuals to become and remain motivated to contribute. It is necessary to regularly provide employees with information about the results and celebrate success with them. Employees should be commended and rewarded for achieved results. This will ensure that the “eyes and ears” in production process will be directed to identification of irregularities and inconsistencies.

### 3.7. Checking System Performance

After implementing selected measures and activities in accordance with the action plan, the question is: “Do implemented measures accomplish anticipated?” To answer the question there must be an appropriate system of measurement, data collection, and data analysis. This system for checking performance involves measuring, monitoring, and targeting and defining corrective actions is critically important for the successful implementation of EEMS.

#### 3.7.1. Measuring and Data Collection

Monitoring the performance of the system begins with measuring and collecting data (from measuring instruments, bills, data on type, and volume of production, etc.). The success of the procedure depends on the quality of data collected. Data to be collected refer to the entire industrial plant or ECC.

Measurement and data collection and analysis of system performance should be systematically organized on the “lower level,” that is, level of ECCs. The care should be taken to avoid overlap or multiple measurements of performance indicators. While developing a plan for measuring equipment installation, the costs of its installation and maintenance should be lower than expected earnings resulting from measurement and data collection.

#### 3.7.2. Comparison of Performance Indicators

Significant activities of EEMS are a regular periodic measurement, analysis, and reporting on performance indicators of the system. These procedures allow energy and environmental managers and other competent employees with appropriate authority to monitor energy and environmental efficiency over time and to compare the efficiencies with the efficiencies of companies with similar production activities. Monitoring environmental performance indicators focuses on ensuring compliance with regulatory requirements regarding environmental protection and related costs, while monitoring energy efficiency indicators focuses on direct cost savings through more efficient use of energy resources.

It is necessary, whenever possible, to compare determined performance indicators of the company or ECCs with the indicators of companies from similar industry, with similar production program or production volume, and so forth. At the international level, there is a relatively large number of benchmarking databases that contain data on specific energy consumption in various industrial sectors, facilities, or processes (EU—BESS [15, 21], Asia-Pacific region—APERC [22], USA—IAC Database [23], etc.). Data on specific energy consumption in the production of wood furniture can be found in some of the databases so the situation in a company can be compared with relevant international experience in the sector.

#### 3.7.3. Monitoring and Targeting

Monitoring and targeting are the essential part of EEMS in an industrial enterprise. These technique uses regularly collected data on energy and environmental performance indicators for

- assessment of company's energy and environmental performance relative to established objectives and targets, norms of the industrial sector, efficiency changes in time and
- systematic checking of compliance with relevant legal and other requirements.

There are three problems that commonly occur when collecting data:

1. insufficient data (data should be recorded at regular intervals),
2. too much data (it is difficult to effectively manage and analyze too much data, too frequently obtained by measuring, and collecting data at regular intervals—e.g., every half hour or more often),
3. incompatible data (data relating to the same interval of time should be compared).

All the benefits of monitoring and targeting arise from the effective analysis of collected data. When the analysis is ineffective, the whole system is weak. A wide range of statistical tools and techniques are available for data analysis: graphical methods (scatter graph, bar charts, Hi-Lo charts, Pareto charts, etc.), measurement of spread (standard deviations, correlation coefficient, fiducial or confidence limits), algebraic regression (regression, least squares, multivariate regression), residual analysis, CUSUM (cumulative sum of differences), control charts, and so forth [14]. Appropriate software applications for monitoring and targeting exist, although standard PC spreadsheet software is suitable for most applications.

After data collection, the next stage is to determine an initial target (base) against which the monitored data will be compared so actual energy consumption (waste generation) can be compared with target. Since the production volume affects the energy consumption (the amount of generated waste), the targeted value cannot be single; it must be related to the production volume. Initial target line can be determined by analysis of historical data on energy consumption (the amount of generated waste). The data on a monthly basis from the previous period (a minimum of one, preferable two years) may be taken as the base data against which future improvements can be assessed. In case that relevant data on energy consumption (waste generation) are unknown, design consumption and the legal allowed amount of waste are considered as an initial target. The target can be later modified as more data are collected and more experience is gained about the system.

**Figure 6:**
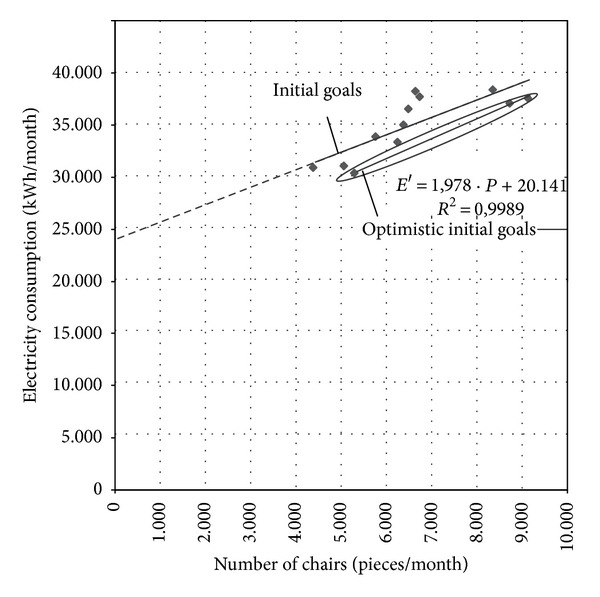
Identifying base (target) line.

Collected data on energy consumption (the amount of generated waste) is displayed as a function of production volume in the appropriate graphical form. From the scatter diagram a base (target) line can be determined (Figure 6).

**Figure 7:**
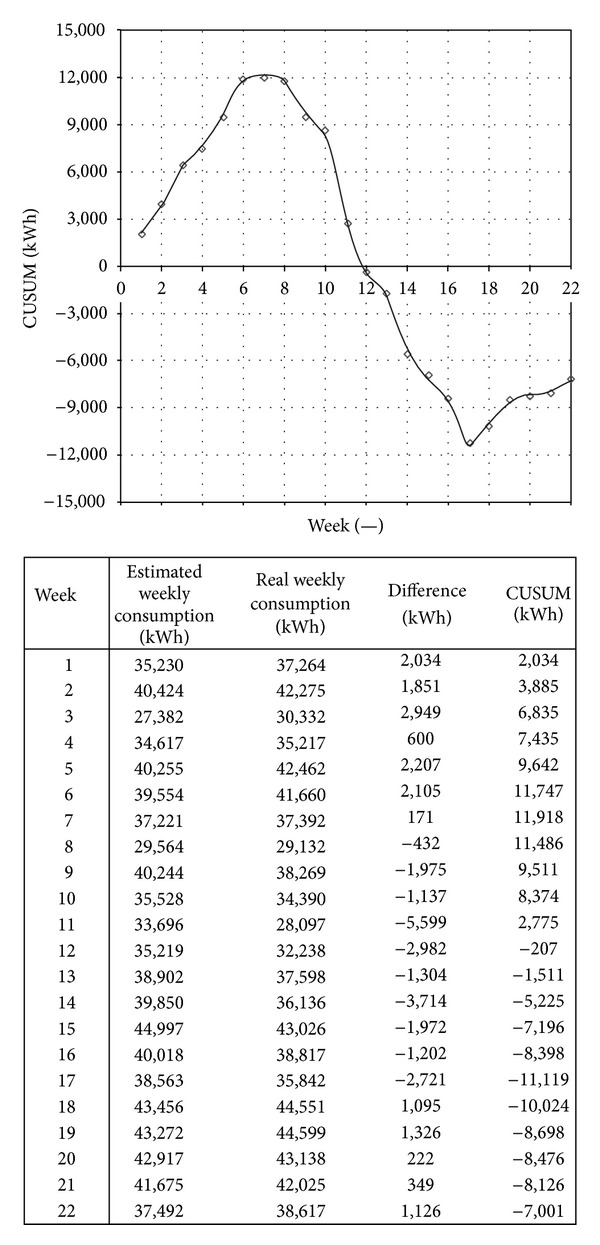
Example of CUSUM application in wood industry.

After setting the initial goals, the company and its ECCs should be constantly monitored to see whether the set goals are achieved. The best way to determine the state of the company is to compare its actual performance with established goals or legal constrains. The method of quantitative estimation of savings or losses by calculating “cumulative sum of the difference”—known as the CUSUM method is the simplest and most effective way of monitoring performance against targets [24]. CUSUM is a simple but very powerful statistical method which points to even small differences in energy and environmental performance (Figure 7). Regular application of this procedure provides energy and environmental managers and accompanying team to quickly recognize any changes in the system.

#### 3.7.4. Benchmarking Performance Indicators

At this stage of EEMS development, the energy and environmental performance of an industrial organization is well known. So, it is the time to develop and maintain company procedures for regular periodic EEMS reviewing in order to determine whether the plans, activities, and procedures are implemented in the required way. This involves answering questions:

- Are the expected objectives achieved?
- Are the plans and procedures established by the company followed?
- Can determined procedures and plans achieve stated objectives of energy and environmental management?
- Is the EEMS effectively implemented and maintained?

It is important that key elements are reviewed and evaluated regularly, at intervals that are defined by the company (at least once a year), either through internal or external verification of EEMS. Audit results should be available to employees with the appropriate level of obligations, so they should timely take corrective action for all detected inconsistencies.

#### 3.7.5. Corrective and Preventive Actions

Any deviation from the requirements of EEMS can be corrected by applying corrective and preventive measures. This helps to ensure adherence of company to continuous improvements of energy and environmental policy. The company should develop, implement, and maintain documented procedures for corrective and preventive actions.

Corrective and preventive actions should lead to improvements in systems based on:

- results of audits (internal and external),
- results of conformity assessment,
- failure to achieve specific objectives in monitoring and measurement process,
- failure to comply with operational control procedures,
- failure to meet target dynamics relative to energy and environmental management.

### 3.8. Management Review

It is necessary to regularly review the introduced EEMS to ensure its continuous and proper functioning. Possible improvements can be identified during these reviews with the ambition to prepare a set of corrective actions in the next step of energy and environmental management cycle.

Since EEMS is a continuous process, each element must achieve the required performance in order to obtain smoothly operation of the whole process. Inadequate implementation of one step may affect other elements and the performance of the whole system. Therefore, the review of EEMS is vital for continuous improvement of the system.

Top management must review and evaluate EEMS to ensure its continual suitability, adequacy, and effectiveness. The management should pay attention to the possible need for changes in EEMS to identify topics where improvements can be made.

To ensure that recommendations are taken into account, the management review must be documented. Team for EEMS implementation has to comply with defined actions and to designate persons responsible for implementing the actions.

## 4. Conclusion

As energy costs continue to rise, industrial plants (even those of low energy industries such as wood furniture industry) need effective way to reduce the amount of consumed energy. Besides, there are a number of economic, environmental, and social reasons why a company should consider waste minimisation initiatives.

Introduction of energy and environmental management system to furniture industry brings to the new “greening of industry” approach. It is a comprehensive and systematic approach for energy conservation and environmental protection efforts in an industrial organization.

According to results presented in [25], the existence of management strategies encourages and determines the innovation tendency of wood furniture companies. Therefore, the improvements in energy and environmental efficiency can bring innovative approach to wood based furniture industry.

## Figures and Tables

**Table 1:** Typical waste streams from the wood furniture industry.

Solid Wastes	Air Emissions	Water Emissions
(i) Sawdust	(i) Solvents from coating operation	(i) Boiler blowdown
(ii) Wood scraps	(ii) Solvents from stripping operation	(ii) Cooling tower blowdown
(iii) Sanding dust	(iii) Solvents from cleaning operation	(iii) Waterwall spray booth blowdown
(iv) Boiler ash	(iv) Wood dust	(iv) Air compressor condensate
(v) Packing materials (boxes, crates, etc.)	(v) Boiler combustion gases	(v) Plating tank rinse water
(vi) Empty containers	(vi) Glues	(vi) Rag washwater
(vii) Maintenance materials		
(viii) Spray booth solids or liquids and filters		
(ix) Spray gun cleaning solutions		
(x) Stripping solids and solutions		
(xi) Off-spec finishing materials		
(xii) Dip tank solids		
(xiii) Spills/contaminated dirt and so forth		
(xiv) Rags from wipe down		

**Table 2:** Energy and environmental management matrix.

Level	Energy and environmental policy	Organising	Staff motivation	Tracking, monitoring, and reporting systems	Staff awareness/training and promotion	Investment
4	Active commitment of top management. Energy management and environmental performance fully integrated into management structure	Clear delegation of responsibility for energy consumption and environmental performance	Formal and informal channels of communication regularly exploited by energy and environmental managers and staff at all levels	Comprehensive system sets targets, monitors consumption, identifies faults, quantifies savings, and provides budget tracking	Marketing the value of energy efficiency and environmental management practice both within the organisation and outside it	Positive discrimination in favour of energy saving and “green” schemes

3	Formal policy, but no active commitment from top management	Energy and environmental managers accountable to appropriate committee	Energy and environmental committee used as main channel together with direct contact with major users	Monitoring and targeting reports for individual premises based on submetering, but energy savings and waste reductions not reported effectively to users	Program of staff training, awareness, and regular publicity campaigns	Cursory appraisal of new building, equipment, and refurbishment opportunities

2	Unadopted policy set by energy and environmental managers	Designated energy and environmental managers, reporting to ad hoc committee, but line management and authority unclear.	Contact with major users through ad-hoc committee chaired by senior departmental manager	Monitoring and targeting reports based on supply meter data	Energy and environmental unit have ad-hoc involvement in budget setting. Some ad-hoc staff awareness and training	Investment with short term pay back only

1	An unwritten set of guidelines. Energy and environmental management the part-time responsibility of someone with limited authority and influence	Informal contacts between energy and environmental managers and a few users	Cost reporting based on invoice data	Managers compile reports for internal use within technical department	Informal contacts used to promote energy and environmental efficiency	Only low-cost measures taken

0	No explicit policy. No managers or any formal delegation of responsibility	No contact with users	No information system	No accounting for energy consumption and waste generation	No marketing or promotion of energy and environmental efficiency	No investment in increasing energy and environmental efficiency

**Table 3:** Energy and environmental policy in XY company (wood furniture producer).

(1) Policy	
The policy of our company is to manage energy and environmental issues in order to	
(i) Buy electricity, water, and wood material at the most economic costs to avoid unnecessary expenditure	
(ii) Improve cost-effectiveness, productivity, and working conditions	
(iii) Protect the workspace and environment	

(2) General objectives	
The long-term objectives are to	
(i) Buy electricity, water, and wood material at the most economic costs	
(ii) Use them as efficiently as possible	
(iii) Reduce the amount of pollution, particularly soil pollution and greenhouse gas emissions (caused by our energy consumption)	
(iv) Reduce wherever possible our dependence on fossil fuels through the use of our waste biomass	

(3) Immediate aims	
In the short term, immediate aims are to	
(i) Gain control over energy and environmental aspects of the business by reviewing and improving metering, operation, maintenance, motivation and training practices,	
(ii) Improve energy and environmental efficiency continuously by implementing effective energy and environmental management programs that support all operations at customer satisfaction while providing a safe and comfortable work environment.	

(4) Action plan	
During the coming years, the following activities will be prepared and undertaken	
(i) Program of housekeeping and maintenance	
(ii) Detailed timetable with specified milestones	
(iii) Indication of actions to be undertaken by designated personnel	
(iv) Promotion plan for raising awareness among employees	
(v) Education of employees about how to save energy at work and at home	
(vi) Review and extra promotional campaign	
(vii) Devise plans for monitoring and evaluating achievements	
(viii) Keep employees fully informed about achievements	
(ix) Establish a reward system to recognize efforts of individuals and groups of employees	
Our aim is to reduce energy and environmental compliance expenditures by a minimum of 5% each year over the next three financial years.	
This policy shall apply to all XY facilities, production units, and employees.	
Date and place	
Signature: ___________________	

**Table 4:** Annual balances of energy and water in a wood furniture industry company.

Fuel	Consumption	Unit	Equivalent energy		Cost	
			kWh	%	€	%
Electricity	388,604	kWh	330,640	77	32,001	94
Biomass/waste	40,050	kg	100,125	23	—	
Water	4,174.5	m^3^			1,934	6

Total			354,265	100		100

**Table 5:** Characteristics of wood waste generated in a furniture industry company [26].

Wood waste	MFC (melamine face board/particle boards)	MDF (medium-density fibreboard)	HDF (high-density fibreboard)
Density (kg/m^3^)	694.5	715.5	879
Lower heating value (MJ/kg)	16,479	17,413	19,401
Amount of the waste (m^3^/year)	115.36	16.27	7.23
Energy content of the waste (GJ/year)	1,320,257	202,708	123,297
Total energy content (GJ/year)	**1,646,262**		

**Table 6:** Opportunities and measures for energy savings in wood furniture industry [27].

Electricity	General measures	(i) Switching off equipment when not in use
		(ii) Load management and demand control
		(iii) Power factor correction
		(iv) Replacement of old transformers
		(v) Tariff system
	Electric motors	(i) Using efficient belt drives
		(ii) Replacing old and using high efficiency motors
		(iii) Replacing overrated motors
		(iv) Variable speed drives
	Lighting	(i) Replacement and use of efficient lamps, ballasts and luminaries
		(ii) Lighting controls
		(iii) Using daylight
		(iv) Lighting maintenance
	Compressed air	(i) COMPRESSED AIR PRODUCTION AND DISTRIBUTION (selection of a compressor and adequate control system, adjusting working pressure, heat recovery from hot compressed air, choosing proper location for installing a compressor, sizing of the compressor suction line, air dryer selection, equipment maintenance)
		(ii) COMPRESSED AIR DISTRIBUTION (leakage prevention and minimization, proper design, and selection of receivers, proper dimensioning of lines)
		(iii) COMPRESSED AIR USAGE (prevention of improper usage, filtration and lubrication, equipment maintenance, usage of efficient air guns)
	Dust collection and transport system	(i) Proper design and construction of hoods-control of air velocity in the pipe
		(ii) Proper sizing of pneumatic pipelines, selection of standard system components, and proper location of their placement
		(iii) Balancing pneumatic network lines
		(iv) Replacing the overrating fans
		(v) Selecting high efficiency fans
		(vi) Improving the energy efficiency of fan's electric motor drive
		(vii) Leakage repair
		(viii) Proper maintenance of components (motors and fans, separators, etc.) and whole system (leakage prevention)
Heat	Boilers (heat generation)	(i) Proper boiler sizing
		(ii) Proper selection and construction of the system for the supply of waste wood material
		(iii) Proper selection and construction of a firebox
		(iv) Control of combustion process (control and reduction of excess air, proper mixing of fuel and air, control of the combustible gas residence time), improved boiler insulation,
		(v) Boiler maintenance
		(vi) Reducing heat loss in flue gases (minimizing flue gas outlet temperature, minimizing the amount of flue gas output, reducing air infiltration)
		(vii) Regeneration of waste heat from flue gasses (using economizers)
		(viii) Condensate return (steam systems)
		(ix) Blow-down heat recovery and so forth
	Hot water or steam distribution	(i) Insulation improvement
		(ii) Insulation maintenance
		(iii) Steam tarp replacement and maintenance (steam systems)
		(iv) Leakage reduction
		(v) Recovering flash steam (steam systems)
		(vi) Electronic control and so forth
	Heat use—space heating	(i) Adjustment and control of heating temperature
		(ii) Building insulation
		(iii) Compressor waste heat usage
	Heat use—process heating (dryers)	(i) Controlling drying process parameters
		(ii) Controlling the amount and distribution of timber in a dryer
		(iii) Installation of a recuperator in the ventilation system
		(iv) Insulation of kilns and so forth

**Table 7:** Measures for waste reduction in wood furniture industry [27, 28].

Operation	Measure
Lumber receiving, drying, and storage	(i) Proper and efficient planning and organizing lumber purchasing and delivering
	(ii) Inspection and sorting of lumber
	(iii) Lumber separation by kiln sticks when stacking
	(iv) Improving boiler efficiency
	(v) Improving drying kiln efficiency
	(vi) Using energy efficient drying techniques
	(vii) Storage of material in a warehouse

Rough end and gluing	(i) Removing defects from rough lumber efficiently
	(ii) Finger jointing (joining of two short sticks or boards)
	(iii) Recycle wood waste and sawdust
	(iv) Using the proper glue and proper gluing techniques

Machining and sanding, assembly	(i) Recycle wood waste and sawdust
	(ii) Adequate designing of wood dust collection and transport system
	(iii) Regular cleaning sanding belts and machine tools
	(iv) Using the proper glue and proper gluing techniques

Finishing	(i) Using alternative coating materials
	(ii) Application of more reliable and more efficient coating application technologies
	(iii) Adequate planning and organizing of production operations and maintenance

Packing, shipping, and warehouse	(i) Enhancing packaging performance by evaluating damage history
	(ii) Enhancing packing performance by evaluating packaging water resistance
	(iii) Decreasing toxic metals content of packaging materials
	(iv) Eliminating ozone depleting substances in packaging materials
	(v) Redesigning packaging to minimize volume and weight—by evaluating packaging materials and closure methods
	(vi) Developing reusable containers
	(vii) Improving compatibility of packaging materials for recycle
	(viii) Recycling other wastes produced in the packaging, shipping, and warehouse

Workspace and equipment maintenance	(i) Using synthetic lubricating oils with longer life and their recycling
	(ii) Maintaining of kilns and their proper control
	(iii) Oil cleaning up with recyclable absorbents
	(iv) Keeping chemical wastes segregated
	(v) Separating and recycling paper, wood, metals, and glass
	(vi) Using wood boiler ash as a soil conditioner

